# *Babesia hegotelforum* sp. nov., a zoonotic *Babesia* species previously referred to as *Babesia sp*. *MO1*

**DOI:** 10.1080/22221751.2026.2637280

**Published:** 2026-03-06

**Authors:** Pallavi Singh, Karel Estrada, Luis Miguel Gonzalez, Ricardo Grande, Sergio Sánchez-Prieto, Emmanuel Cornillot, Omar Harb, Alejandro Sanchez-Flores, Estrella Montero, Karine G. Le Roch, Stefano Lonardi, Choukri Ben Mamoun

**Affiliations:** a Department of Internal Medicine, Section of Infectious Diseases, Yale School of Medicine, New Haven, CT, USA; b Department of Microbial Pathogenesis, Yale School of Medicine, New Haven, CT, USA; c Department of Pathology, Yale School of Medicine, New Haven, CT, USA; d Unidad Universitaria de Secuenciacion Masiva y Bioinformatica, Instituto de Biotecnologia, Universidad Nacional Autonoma de Mexico, Cuernavaca, Mexico; e Laboratorio de Referencia e Investigación en Parasitología, National Center for Microbiology, Instituto de Salud Carlos III, Majadahonda, Spain; f Institut de Biologie Computationnelle (IBC), and Institut de Recherche en Cancérologie de Montpellier (IRCM -INSERM U1194), Institut régional du Cancer Montpellier (ICM) & Université de Montpellier, Montpellier, France; g Department of Biology, University of Pennsylvania, Philadelphia, PA, USA; h Department of Molecular, Cell and Systems Biology, University of California, Riverside, CA, USA; i Department of Computer Science and Engineering, University of California, Riverside, CA, USA

**Keywords:** Human babesiosis, *Babesia hegotelforum*, Tick-borne disease, Zoonosis, comparative genomics

## Abstract

A zoonotic *Babesia* species previously referred to as *Babesia sp*. *MO1* is formally described and named here as *Babesia hegotelforum sp. nov*. This taxon is distinct from *Babesia divergens* based on genome-wide sequence divergence, phylogenetic placement, host associations, and clinical presentation. The parasite infects erythrocytes of humans, and eastern cottontail rabbits (*Sylvilagus floridanus*), and is transmitted by *Ixodes dentatus*. The holotype consists of a Giemsa-stained thin blood smear and cryopreserved infected erythrocytes from the cloned isolate BML-*Bh*-B12 at ≤10 passages in continuous in vitro culture. Paratype material includes five additional clones (BML-*Bh*-H1, BML-*Bh*-F12, BML-*Bh*-H6, BML-*Bh*-A3, and BML-*Bh*-F1) derived from BEI Resources strain NR-50441, along with the original mixed isolate NR-50441. This species description meets the requirements of the International Code of Zoological Nomenclature and establishes *Babesia hegotelforum* sp. nov. as a distinct species of clinical and epidemiological significance in North America.

## Introduction

*Babesia* sp. *MO1* was first recognized in a fatal case of human babesiosis in the state of Missouri in the United States in 1996, in a splenectomized patient without travel history to regions endemic for *Babesia divergens* [1]. Although the organism was initially characterized based on the 18S small subunit rRNA (18S rRNA) sequence similarity to *B. divergens* [1], the clinical, epidemiological, and ecological features of this infection differed from European *B. divergens* babesiosis [1]. Blood from the patient failed to cause infection in Holstein-Friesian calves, which are hosts for the European *B. divergens*, and inoculated animals remained fully susceptible upon challenge inoculation with *B. divergens* [2]. Subsequent investigations identified the same organism in wildlife, establishing its presence in North America [3]. *B. divergens* appears to be a global species complex comprised of at least 3 lineages (EU, U.S., and Asia) [4]. The U.S. lineage is represented by *Babesia sp. MO1*.

The Missouri index case had hunted rabbits prior to his illness (EJ Masters, personal communication to SR Telford, 2001). Enzootic transmission studies demonstrated the presence of this parasite in the eastern cottontail rabbit (*Sylvilagus floridanus*) and implicated *Ixodes dentatus* as the vector [3]. An isolate from Nantucket rabbits was propagated in vitro, and failed to infect cattle, demonstrating that *MO1* was not *B. divergens* [5].

The organism was later isolated and adapted to continuous in vitro culture in human erythrocytes [5], resulting in the strain now maintained as BEI Resources NR-50441. Clonal derivatives of this isolate have been generated and characterized, permitting reproducible study of its morphology and growth characteristics [6].

Subsequent cases include a reported human babesiosis case in Kentucky [7], and a transfusion acquired case reported from an Arkansas resident, with reference to another PCR positive sample from a Missouri patient in 2010 [8]. Collectively, these reports established this organism as a distinct cause of human babesiosis in North America, independent of *B. microti* and *B. duncani*. Clinically, infection is indistinguishable from *B. divergens* babesiosis; however, the underlying biology of this agent differs substantially from *B. divergens* and from classical *B. divergens* infections.

Recent whole-genome sequencing, comparative genomics, and phylogenomic analyses demonstrate that *B. hegotelforum* forms a lineage distinct from *B. divergens* sensu stricto [6]. Average nucleotide identity (ANI) between *B. hegotelforum* and *B. divergens* isolates ranges between 96.7 and 96.8%, whereas ANI values among *B. divergens* strains exceed 99.1% (Table 1), consistent with species-level separation [6]. Genome-wide supertree phylogenomic reconstruction based on 2,381 orthologous loci consistently placed *B. hegotelforum* sp. nov. as a well-supported, independent lineage within *Babesia* sensu stricto, with >99% concordance across reconstructed trees [6]. Genome organization in *B. hegotelforum* is broadly conserved relative to *B. divergens*, including chromosome number (three molecules in the haploid nuclear genome), overall genome size (∼11 Mbp), and the structure of the conserved coding core [6]. However, major differences are observed in the composition and organization of multigene families and in the subtelomeric regions of the chromosomes [6]. Notably, the largest lineage-specific multigene family, UMGF1, comprises approximately 30 genes in *B. hegotelforum*. Approximately 300 orthologous genes exhibit substantial sequence divergence between *B. hegotelforum* and *B. divergens*, with patristic distances comparable to those observed between distinct *Babesia* species. These genome-wide differences, together with distinct reservoir host association, vector specificity, intraerythrocytic developmental characteristics, and phylogenomic placement, support formal recognition of this organism as a new species under the International Code of Zoological Nomenclature (ICZN).

**Table 1.** Diagnostic features distinguishing *B. hegotelforum* sp. nov. from related zoonotic *Babesia* species.

Feature	***B. hegotelforum***	***B. divergens***	***B. duncani***	***B. microti***
**ANI of *B. hegotelforum* vs**	100%	∼96.7–96.8%	<95%	<90%
**Phylogenetic clade**	*Babesia* sensu stricto., (Clade VI)	*Babesia* sensu stricto. (Clade VI)	Clade II	Clade I
**Primary reservoir**	Cottontail rabbit (*Sylvilagus floridanus*)	Cattle (*Bos taurus*)	Mule dear (*Odocoileus hemionus*)	Rodents (*Peromyscus leucopus*)
**Vector**	*Ixodes dentatus*	*Ixodes ricinus*	*Dermacentor albipictus*	*Ixodes scapularis*
**Multigene families (MGFs)**	290 *vesa* genes 10 Unique MGFs (with at least 3 members) (PMID: 39148308)	134 *vesa* genes (PMID: 39148308)	73 UMGFs 105 OMGFs (PMID: 37055610)	BMN-family Tpr-like Vesa-like (PMID: 22833609)

## Materials and methods

### Adaptation and continuous in vitro culture of *Babesia hegotelforum*

The *Babesia hegotelforum* isolate used for species description corresponds to BEI Resources strain NR-50441, originally obtained from an eastern cottontail rabbit (*Sylvilagus floridanus*) collected on Nantucket Island, Massachusetts [2]. The initial ex vivo adaptation and continuous in vitro culture of *Babesia* sp. *MO1* were first established by Spencer et al. [5], who demonstrated successful replication of the parasite in human red blood cells and a strict host erythrocyte specificity distinct from *B. divergens*. A cryovial including infected blood received from BEI Resources was thawed (detailed protocol in supplemental methods) and propagated continuously *in vitro* culture in A^+^ human erythrocytes using RPMI1640 medium supplemented with 0.5% Albumax II under microaerophilic conditions (2% O_2_, 5% CO_2_, 93% N_2_) at 37°C. Parasitemia was monitored daily by examination of Giemsa-stained blood smears using light microscopy. Between 5,000 and 10,000 total RBCs were counted to determine parasitemia percentage. Cultures were sub-passaged by dilution into fresh human erythrocytes at regular intervals (every 5th or 6th day) to maintain optimal growth.

Six clonal lines were generated from the parental isolate by three rounds of limiting dilution at early passage (Table 2), as described previously for other parasites such as *B. duncani* [6], and their molecular identity was confirmed by pulsed-field gel electrophoresis as well as genomic sequencing for at least two clones (BML-*Bh*-B12 and BML-*Bh*-F12) [6]. These clonal derivatives form the basis of the material designated as holotype and paratypes in the present species description.

**Table 2.** Clonal lines of *B. hegotelforum* derived from BEI NR-50441.

Clone id	Derivation method	Passage at cloning
**BML-Bh-B12**	Limiting dilution	≤10
**BML-Bh-H1**	Limiting dilution	≤10
**BML-Bh-F12**	Limiting dilution	≤10
**BML-Bh-H6**	Limiting dilution	≤10
**BML-Bh-A3**	Limiting dilution	≤10
**BML-Bh-F1**	Limiting dilution	≤10

A complete, step-by-step protocol for continuous in vitro culture and clonal derivation of *B. hegotelforum* is provided in the Supplementary Methods.

Genomic sequencing data generated for this study are publicly available through NCBI under Bioproject accession number PRJNA1032622. Phylogenetic relationships were inferred using genome-wide alignments and phylogenomic trees generated as part of the multi-omics and comparative genomic analysis of this taxon reported by Singh and colleagues [6].

### Description

**Figure 1.**
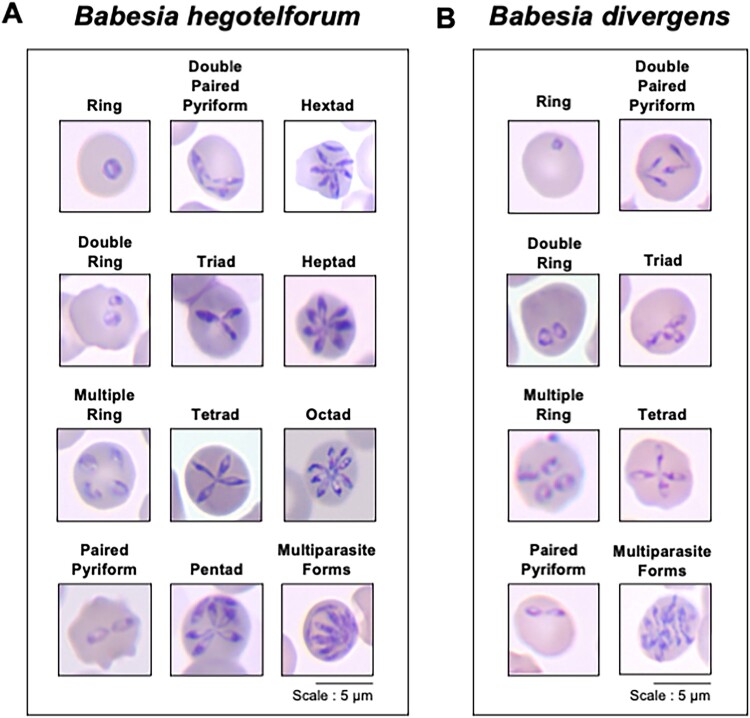
Comparative morphology of intraerythrocytic stages of *Babesia hegotelforum* and *B. divergens* observed by Giemsa staining. A. Representative light microscopy images showing characteristic developmental stages of *B. hegotelforum* (A) and *B. divergens* (B) in human erythrocytes in vitro.

Erythrocytic stages of *Babesia hegotelforum* sp. nov. include ring-like trophozoites, paired pyriforms, tetrads and multi-merozoite forms exhibiting a distinctive flower-like configuration of five, six, seven or occasionally eight merozoites (Figure 1). These developmental forms are commonly observed during continuous in vitro culture [6]. In contrast, *B. divergens* typically produces tetrads consisting of four merozoites joined at a central point, and a higher number of merozoites within a single erythrocyte generally arise through multiple independent invasion events rather than through the formation of multinucleated structures (Figure 1) [9]. Although some morphological overlap exists between the two species, the relative frequency and organization of multi-merozoite forms differ under comparable in vitro conditions.

*B. hegotelforum* parasites are strictly intraerythrocytic and lack detectable pigment. Infected erythrocytes may vary mildly in shape but are not consistently enlarged. Under standard in vitro culture conditions, parasitemia typically reaches maximal levels of approximately 8-10%, which is lower than that commonly observed for *B. divergens* in continuous human erythrocyte cultures.

The nuclear genome of *B. hegotelforum* consists of three chromosomes and has an overall size of approximately 11 Mbp, comparable to that of *B. divergens* isolates [6]. Chromosome length polymorphism (CLP) was observed in chromosomes I and II among the holotype and paratype clones, as demonstrated by pulsed-field gel electrophoresis (PFGE) [6]. Differences at the extremities of chromosome III between the B12 and F12 clones further suggest that CLP may also affect this chromosome, similar to patterns reported for *B. divergens* [6]. Size variation was primarily associated with subtelomeric regions. Despite these polymorphisms, overall chromosomal synteny within the conserved coding core is maintained between *B. hegotelforum* and *B. divergens*. A notable exception is a large insertion present in chromosome I of *B. divergens* that is absent in *B. hegotelforum*, further supporting genomic divergence between the two taxa [6].

### Diagnosis

*Babesia hegotelforum* sp. nov. is distinguished from *B. divergens*, *B. odocoilei*, *B. duncani*, and *B. microti* by a combination of genome-wide divergence, phylogenetic placement, host and vector associations, erythrocytic developmental patterns, and genomic organization [6]. ANI values between *B. hegotelforum* and *B. divergens*, which are between 96.7% and 96.8%, fall well below those observed among *B. divergens* strains (>99%). Comparative analyses identified 637 proteins unique to *B. hegotelforum*, including lineage-specific multigene families and a divergent repertoire of variant erythrocyte surface antigen (*vesa*) genes [6].

### Holotype

Holotype: Giemsa-stained thin blood smear and cryopreserved *Babesia hegotelforum* sp. nov.-infected erythrocytes from clone BML-Bh-B12 at ≤10 passages in continuous in vitro culture, derived from BEI Resources isolate NR-50441. This clone is available from the Ben Mamoun lab at Yale upon request, and its deposition with BEI Resources is currently underway.

### Paratypes

Paratypes: Clones BML-*Bh*-H1, BML-*Bh*-F12, BML-*Bh*-H6, BML-*Bh*-A3, and BML-*Bh*-F1 (Table 2), derived from BEI Resources isolate NR-50441. Historical paratype: BEI Resources NR-50441 original isolate. These paratypes are available from the Ben Mamoun lab at Yale upon request, and their deposition with BEI Resources is currently underway.

### Etymology

The species epithet hegotelforum honors Drs. Barbara Herwaldt, Heidi K. Goethert and Sam R. Telford III, in recognition of their pioneering contributions to ecological characterization, and early transmission studies of this parasite.

### ICZN compliance statement

This species description conforms to Articles 8–16 of the International Code of Zoological Nomenclature. Holotype and paratype materials are publicly accessible in recognized biological repositories.

## Supplementary Material

Supplementary Methods.docx
