# Supplementary material for: Babesia hegotelforum sp. nov., a zoonotic Babesia species previously referred to as Babesia sp. MO1
Source: Emerg Microbes Infect. 2026 Mar 6;15(1):2637280. doi: 10.1080/22221751.2026.2637280 (PMC12973771; doi:10.1080/22221751.2026.2637280)
Supplement: Supplementary Methods.docx [file TEMI_A_2637280_SM7024.docx]

**Supplementary Methods**

**1. Thawing, adaptation and continuous in vitro culture of *Babesia hegotelforum*.**

**a) Parasite sources**

The parental mixed isolate NR-50441is available from BEI Resources. Clonal lines (BML-*Bh*-A3, BML-*Bh*-B12, BML-*Bh*-F1, BML-*Bh*-F12, BML-*Bh*-H1, and BML-*Bh*-H6) can be obtained from the Ben Mamoun laboratory upon request.

**b) Thawing procedure**

Cryovials (NR-50441) from BEI resources or clonal lines from Ben Mamoun laboratory were thawed as follows:

1. Rapidly thaw the cryovial at 37°C water bath for 1-2 minutes.
2. Transfer the contents of the vial (~ 1000 μL) into a 50 mL centrifuge tube.
3. Slowly add 200 uL of 12% NaCl dropwise while gently mixing. Incubate at room temperature for 5 minutes.
4. Add 9 mL of 1.6% NaCl dropwise (~10x of the original cryovial volume), gently mix, and centrifuge at 1500 g for 5 minutes.
5. Remove the supernatant and resuspend the pellet in 40 mL of incomplete RPMI1640 medium (incomplete means without any serum or supplements).
6. Centrifuge at 1500 g for 5 minutes. Repeat the wash once more.
7. After the final wash, resuspend the packed erythrocyte pellet in 250 μL of fresh A^+^ human red blood cells (RBCs).
8. Add 4 mL of either complete DMEM/F12 medium (supplemented with 20% fetal bovine serum, 1x of 50x HT Media Supplement Hybrid-Max TM (Sigma, H0137), 1x of 100x (200 mM) L-Glutamine (Gibco, 25030-081), 1x of 100x Antibiotic-Antimycotic (Gibco, 15240-062) and 1x of 100x (10 mg/mL) Gentamicin (Gibco, 15710-072)) in case of NR-50441, or complete RPMI1640 medium (supplemented with 0.5% Albumax II, 1x of 100x (200 mM) L-Glutamine (Gibco, 25030-081), 1x of 100x Antibiotic-Antimycotic (Gibco, 15240-062) and 1x of 100x (10 mg/mL) Gentamicin (Gibco, 15710-072)) for clonal lines.
9. Make blood smears every 24 - 48 hours and stain with Giemsa to monitor parasitemia.

**c) Adaptation and maintenance**

After thawing NR-50441, culture medium (complete DMEM/F12) should be replaced daily, and fresh RBCs should be added every 5^th^ or 6^th^ day. This ensures that cultures contain enough healthy erythrocytes throughout the culture revival and adaptation phase. NR-50441 adapts to in vitro culture slowly, so it is very common to see parasitemia around 1-2% for the first 2-3 weeks after thawing. During this time, it is common to see the predominance of ring stages and paired pyriforms. Beginning around week 4, parasitemia gradually increases, and all intraerythrocytic stages could be observed. The cultures can be maintained continuously to obtain a parasitemia of 8-10%. At this point, the cultures should be diluted back to 1% parasitemia by adding fresh human erythrocytes to maintain 5% hematocrit (HC).

After thawing clonal lines, the culture medium (complete RPMI1640) must also be replaced daily, and fresh RBCs must be added every 5-6 days. Parasitemia should be monitored daily by examination of Giemsa-stained smears. The clonal lines expand more rapidly that the parental isolate. Once parasitemia exceeds 2%, doubling parasitemia is approximately 48-50 hours. The clonal lines can be propagated to a parasitemia of 10-12%. After reaching this parasitemia, the cultures must be diluted to 1% parasitemia at 5% hematocrit for continued propagation.

**2. Dilution cloning of *B. hegotelforum*.**

**Limiting dilution**

In vitro culture of *B. hegotelforum* NR-50441 isolate was initiated in A^+^ human RBCs in complete DMEM/F12 medium at 0.5% parasitemia and 5% hematocrit (HC). The parasites were allowed to expand to 5% parasitemia. The culture was serially diluted to obtain 30 parasites in 20 mL volume at 5% HC. A sample of 200 μL of this parasite suspension was distributed per well of a 96-well plate. Medium was replaced every third day.

**Identification of positive wells**

To determine which wells of the 96-well plate contain parasites, parasitemia estimation was performed using SYBR Green-I based fluorescence assay on day 20 using protocol based as described by Abraham et al., 2018 (PMID: 30463941). A 25 μL sample was collected from each well of the 96-well plate and mixed with 25 μL of SYBR Green-I lysis buffer consisting of:

- 20 mM Tris, pH 7.4
- 5 mM EDTA
- 0.008% saponin
- 0.08% Triton X-100
- 1X SYBR Green-I (Molecular Probes, 10,000X solution in DMSO, Eugene, OR, USA).

Uninfected human RBCs (5% HC, 25 μL volume) served as negative controls.

The SYBR Green-I measurement plates were incubated at 37 °C in the dark for 1 h and read using a BioTek Synergy MX fluorescence plate reader with an excitation of 497 nm and emission of 520 nm. Using readings from uninfected human RBC as a background, the readings for different wells of the cloning plate were calculated to determine which wells of the 96-well plate contained parasites (higher SYBR Green-I readings in comparison to the negative control). After identification of the wells containing parasite clones, Giemsa smears were prepared from the same wells and observed under light microscopy to confirm the presence of the parasites.

**Expansion and cryopreservation of clones**

Six *B. hegotelforum* clones (BML-*Bh*-A3, BML-*Bh*-B12, BML-*Bh*-F1, BML-*Bh*-F12, BML-*Bh*-H1, and BML-*Bh*-H6) were selected and expanded to 1 mL cultures. The cultured were monitored until parasitemia reached 2%. The clones were further expanded to 10 mL cultures and propagated until they reached 5-6% parasitemia. At this stage, cryovials were prepared and stored in liquid nitrogen. Parallel cultures were maintained continuously in vitro to determine growth rate and conduct other biological assays.
